# iTRAQ analysis of a mouse acute myocardial infarction model reveals that vitamin D binding protein promotes cardiomyocyte apoptosis after hypoxia

**DOI:** 10.18632/oncotarget.23025

**Published:** 2017-12-06

**Authors:** Yun Wu, Fen Liu, Xiang Ma, Dilare Adi, Ming-Tao Gai, Xiang Jin, Yi-Ning Yang, Ying Huang, Xiang Xie, Xiao-Mei Li, Zhen-Yan Fu, Bang-Dang Chen, Yi-Tong Ma

**Affiliations:** ^1^ Xinjiang Key Laboratory of Cardiovascular Disease Research, First Affiliated Hospital of Xinjiang Medical University, Urumqi 830011, P.R. China; ^2^ Department of Cardiology, First Affiliated Hospital of Xinjiang Medical University, Urumqi 830011, P.R. China; ^3^ Department of General Practice, First Affiliated Hospital of Xinjiang Medical University, Urumqi 830011, P.R. China; ^4^ Proteomics Research Center, Ruikangda Medical Research Institution, Urumqi 830063, P.R. China

**Keywords:** acute myocardial infarction, quantitative proteomics, isobaric tags for relative and absolute quantitation, vitamin D binding protein

## Abstract

The proteome profile changes after acute myocardial infarction (AMI) and the roles played by important protein species remain poorly understood. Here, we constructed a mouse AMI model by ligating the left coronary artery of male C57B/6J mice to investigate the molecular changes after AMI on the protein level. Total proteins of the left ventricle were extracted and quantitatively analyzed by isobaric tags using relative and absolute quantitation (iTRAQ) technologies. The transcript and protein levels of important genes were further validated using quantitative polymerase chain reaction and western blot. An oxygen and glucose deprivation/reperfusion cell model was constructed using H9C2 cells to further validate the expression patterns and functions of important proteins after hypoxia. Seven hundred seventy-six proteins were identified as differentially abundant proteins after AMI, of which 406 were accumulated, and 370 were reduced. Gene ontology enrichment analysis showed that the most enriched molecular function category terms were binding, including calcium ion biding, GTP binding, actin binding and lipid binding. The expression levels of vitamin D binding protein (VDBP) and its related proteins were increased in both left ventricular tissue and H9C2 cells after ischemia-hypoxia. Overexpression of VDBP in H9C2 cells reduced vitamin D receptor and promoted the cell apoptosis rate after hypoxia. Our data provided new insights into proteome profile changes after AMI and indicated that VDBP could promote cardiomyocyte apoptosis after hypoxia.

## INTRODUCTION

Acute myocardial infarction (AMI) is a major health problem, due to sudden interrupted blood flow to the heart, leading to a high incidence of heart failure [1]. The World Bank estimated that the number of patients with AMI in China will increase to 23 million by 2030 [2]. Although most patients with myocardial infarction can undergo percutaneous coronary intervention (PCI) and take the drugs recommended by guidelines, the risk of in-hospital mortality did not significantly decrease over the past 10 years [3]. The most important risk factor is left ventricular (LV) remodeling after AMI. As is known, AMI leads to structural and biomechanical changes in the LV, including myocardial apoptosis, collagen deposition, fibrosis, hypertrophy and modifications in the ventricular architecture [4]. Determining the molecular changes responsible for LV remodeling after AMI is currently essential for the cardiovascular research field.

Animal models are an effective approach to investigate the molecular mechanism of AMI. Gao *et al.* ligated the left coronary artery to create a mouse model of AMI, and they found that left ventricular remodeling leading to rupture occurred at 2–6 days after AMI in C57B/6J mice, constituting a main cause of sudden cardiac death after AMI, similar to humans [5, 6].

Vitamin D binding protein (VDBP) elicits a variety of biological functions *in vivo*. In addition to binding to vitamin D in the plasma as a vitamin D transporter, VDBP is also involved in inflammation as an actin scavenging after vascular injury [7]. Gelsolin (GSN), coupled with VDBP, is an actin-modulating protein that binds to actin monomers or filaments, and it was reported to serve as an inhibitor of acute myocardial injury [8]. A proteomics study revealed that the level of VDBP was increased in the serum of ST elevated myocardial infarction (STEMI) patients, reducing the aggregation rate and prolonging coagulation time [9]. However, another study showed that the level of VDBP was decreased in the serum from coronary artery disease patients, markedly in multi-vascular disease patients [10]. More research work is needed to determine the function of VDBP after AMI.

High-throughput LC MS/MS technologies have developed significantly over the last 10 years [11–13]. Isobaric tags for relative and absolute quantitation (iTRAQ) technology has been applied to find potential early biomarkers for Alzheimer’s disease, gastric adenocarcinoma and AMI [14–17]. Our lab successfully constructed an AMI model in C57B/6J mice, as previously reported [18]. Here, we report on a comparative proteomics analysis based on iTRAQ technology conducted to investigate potential biomarkers and to develop a mechanism of acute myocardial infarction using model mice.

## RESULTS

### Successful construction of the mouse acute myocardial infarction model

**Figure 1:**
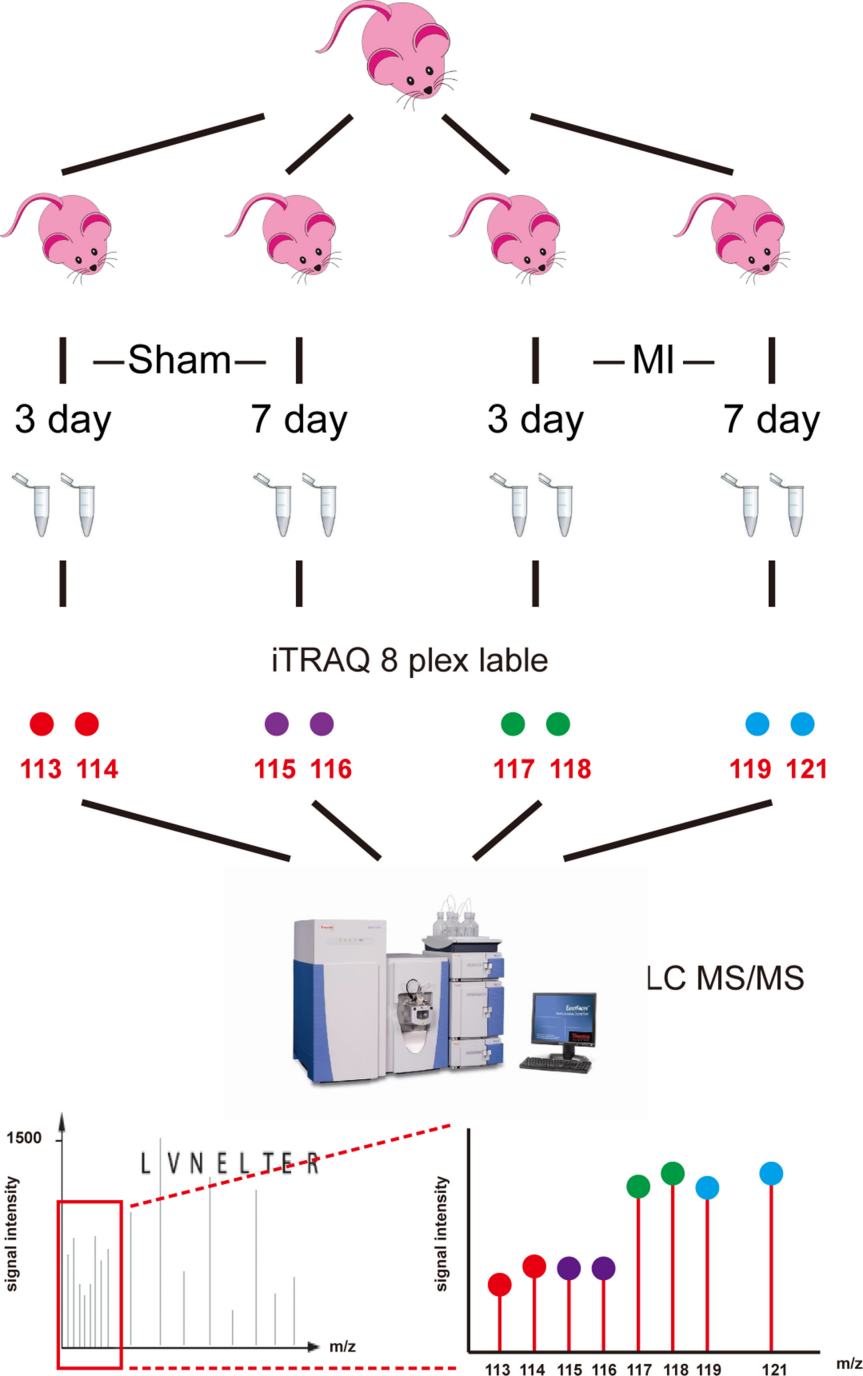
Workflow of iTRAQ analysis of acute myocardial infarction mouse model Left ventricles were obtained from acute myocardial infarction model mice at 3 days and 7 days after surgery (MI-3d and MI-7d) and sham operation (Sham-3d and Sham-7d). Eight independent biological duplicates were used for each group. Pooled protein samples were digested by trypsin. Two technical replicates were performed for each group (isobaric tags 113 and 114 for sham-3d; 115 and 116 for sham-7d; 117 and 118 for MI-3d; 119 and 121 for MI-7d). iTRAQ-labeled peptides were subsequently fractionated by HPLC and then were analyzed by LC-MS/MS.

The workflow of comparable proteomics analysis of the mouse AMI model was shown. Briefly, the left ventricles of mice were obtained at 3 days (MI-3d, Sham-3d) and 7 days (MI-7d, Sham-7d) after surgery. Total proteins were extracted and then digested by trypsin, followed by two-dimensional LC MS/MS analysis. Two independent biological duplications were performed for each group (Figure 1).

Significant cardiac dysfunction was observed after AMI (Table 1, Supplementary Table 1). The left ventricle dimension parameters were significantly increased after AMI (*p* < 0.001), while the systolic blood pressures were significantly decreased after AMI (*P* < 0.01). Hematoxylin and eosin (H&E) staining images of heart tissue samples from C57B/6J mice after surgery and sham operations also indicated that the animal model was successfully constructed (Supplementary Figure 1).

**Table 1:** Echocardiographic and hemodynamic measurements at 3 days (MI-3d) and 7 days (MI-7d) after AMI

Parameters	After 3 d		After 7 d	
	Sham-3d (*n* =10)	MI-3d (*n* =9)	Sham-7d (*n* =10)	MI-7d (*n* =7)
**Echocardiography^$^**				
LVEDd (mm)	3.31 ± 0.28	4.39 ± 0.31^***^	3.32 ± 0.40	5.36 ± 0.50^###^
LVESd (mm)	1.66 ± 0.31	3.45 ± 0.75^***^	1.64 ± 0.28	4.75 ± 0.63^###^
ExLVDd (mm)	4.73 ± 0.40	5.40 ± 0.48^**^	4.64 ± 0.37	6.22 ± 0.41^###^
LVAWs (mm)	1.42 ± 0.12	0.40 ± 0.10^***^	1.35 ± 0.12	0.41 ± 0.23^###^
LVAWd (mm)	0.85 ± 0.16	0.39 ± 0.16^***^	0.79 ± 0.11	0.33 ± 0.14^###^
FS (%)	49.84 ± 8.56	21.71 ± 15.59^***^	49.81 ± 10.49	11.69 ± 5.79^###^
**Hemodynamics^$^**				
HR (beats/min)	344.75 ± 17.47	347.78 ± 29.55	366.22 ± 20.50	343.57 ± 35.99
SBP (mm Hg)	112.13 ± 25.33	84.67 ± 9.99^**^	110.56 ± 18.21	81.57 ± 13.20^##^
DBP (mm Hg)	79.63 ± 17.79	69.33 ± 11.03	74.78 ± 13.74	67.29 ± 11.01
LVSP (mm Hg)	113.75 ± 22.60	92.67 ± 9.98^*^	105.44 ± 16.74	88.86 ± 12.21
+dP/dt (mm Hg/s)	9981.5 ± 2502.60	6243.89 ± 1401.72^***^	8193.78 ± 1821.76	4847.29 ± 1386.83^##^
-dP/dt (mm Hg)	9243.50 ± 2205.78	5607.00 ± 1816.97^***^	8697.78 ± 1703.62	3883.00 ± 728.92^###^

^*^p < 0.05 versus Sham-3d; ^**^p < 0.01 versus Sham-3d; ^***^p < 0.001 versus Sham-3d

^#^*p* < 0.05 versus Sham-7d; ^##^*p* < 0.01 versus Sham-7d; ^###^*p* < 0.001 versus Sham-7d

$, echocardiography parameters; BW, body weight; LVEDd, left ventricular internal end-diastolic diameters; LVESd, left ventricular internal end-systolic diameter; ExLVDd, external left ventricular diastolic diameter; LVAWs, left ventricular systolic anterior wall thickness; LVAWd, left ventricular diastolic anterior wall thickness; FS, fractional shortening. Hemodynamic parameters, HR, heart rate; SBP, systolic blood pressure; DBP, diastolic blood pressure; LVSP, left ventricular systolic pressure; dP/dtmax, maximal rates of rise in left ventricular pressure; dP/dtmin, maximal rates of fall in left ventricular pressure.

### Quantitative proteomic and bioinformatic analyses of AMI model mice

**Figure 2:**
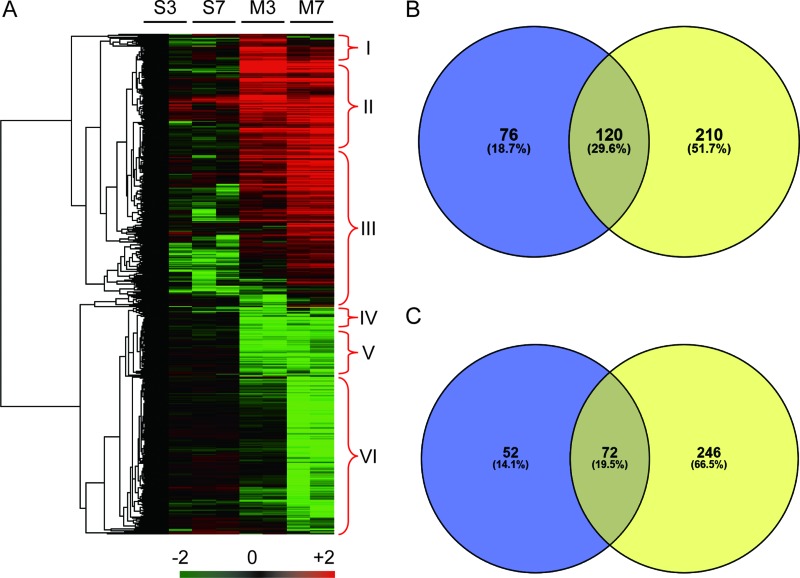
Heat map and Venn diagram of significantly differential abundant proteins (**A**) Heat map of 776 proteins that showed significantly differential accumulation levels in either MI-3d or MI-7d. S3, Sham-3d; S7, Sham-7d; M3, MI-3d; M7, MI-7d. The protein accumulation patterns in cluster I to cluster VI are indicated. (**B**) Venn diagram of proteins preferentially accumulated in MI-3d (blue) and MI-7d (yellow). (**C**) Venn diagram of proteins significantly reduced in MI-3d (blue) and MI-7d (yellow).

A total of 545,987 MS/MS spectra were obtained and matched to 61,818 distinct peptides at a 0.05 *p* value cutoff, corresponding to 2540 detected protein groups (Supplementary Table 2), of which 776 were considered significantly differentially abundant proteins (DAPs, fold change > 2 and *p* value < 0.05). Six sub-clusters were determined: specially accumulated in MI-3d (I), accumulated in both MI-3d and MI-7d samples (II) and specially accumulated in MI-7d (III), specially reduced in MI-3d (IV), reduced in both MI-3d and MI-7d (V) and specially reduced in MI-7d (VI) (Figure 2A). The Venn diagram of DAPs accumulated (Figure 2B) or reduced (Figure 2C) in MI mice were shown. The 196 DAPs (76 MI-3d special DAPs and 120 MI-3d and MI-7d co-DAPs) were considered potential biomarkers for early diagnosis of acute myocardial infarction (Figure 2B and Supplementary Tables 3 and 4). The 210 MI-7d special DAPs were considered to be involved in coagulative necrosis of myocardial fibers and new capillary granulation especially observed in hearts at 7 days after AMI (Figure 2B and Supplementary Tables 5 and 6).

Gene ontology annotations of the 776 DAPs were obtained by Blast2GO [24], and the enriched GO terms were determined by agriGO [25]. The tree views of the enriched GO terms of the molecular function categories showed that the molecular function of binding was the most significantly enriched GO term, including calcium ion binding, GTP binding, actin binding (Supplementary Figure 2A), lipid binding and cytoskeletal protein binding (Supplementary Figure 2B). The enriched biological process category GO terms of the MI-3d and MI-7d special DAPs are listed in Table 2, with most significant pathways being complement activation (GO:0006957) for MI-3d and regulation of actin filament polymerization (GO:0030833) for MI-7d. Further, the enriched biological process category GO terms of proteins accumulated or reduced both in MI-3d and MI-7d are listed in Supplementary Tables 7 and 8, and the tree views are shown in Supplementary Figures 3 and 4, respectively.

**Table 2:** Information of enriched GO terms of MI-3d and MI-7d special accumulated proteins

GO_acc.	GO Terms	No. Proteins	FDR-*p* value
**MI-3d special accumulated proteins**			
GO:0006957	complement activation, alternative pathway	5	4.60E-09
GO:0006412	translation	12	4.60E-09
GO:0006810	transport	17	1.30E-05
GO:0006461	protein complex assembly	6	0.00035
GO:0044057	regulation of system process	5	0.0013
GO:0006873	cellular ion homeostasis	5	0.0034
GO:0010033	response to organic substance	6	0.0051
GO:0006066	alcohol metabolic process	5	0.0051
**MI-7d special accumulated proteins**			
GO:0030833	regulation of actin filament polymerization	9	3.00E-09
GO:0008380	RNA splicing	11	1.90E-06
GO:0016192	vesicle-mediated transport	16	3.60E-06
GO:0031589	cell-substrate adhesion	8	7.70E-06
GO:0006397	mRNA processing	11	2.00E-05
GO:0030198	extracellular matrix organization	7	0.00013
GO:0019725	cellular homeostasis	12	0.0004
GO:0019752	carboxylic acid metabolic process	12	0.002
GO:0051260	protein homooligomerization	5	0.0026
GO:0006486	protein amino acid glycosylation	5	0.0034
GO:0007242	intracellular signaling cascade	16	0.0039
GO:0006886	intracellular protein transport	8	0.0062
GO:0001568	blood vessel development	8	0.0078
GO:0043066	negative regulation of apoptosis	8	0.009

### Validation of vitamin D binding protein expression patterns

To validate the transcript expression levels and protein levels of representative DAPs, vitamin D binding protein (VDBP) and its related proteins were chosen from among the most significant GO terms (GO:0003779, actin binding, Supplementary Figure 2). The mRNA expression level of VDBP and two related genes, vitamin D receptor (VDR) and gelsolin (GSN), were significantly increased in both MI-3d and MI-7d (Supplementary Figure 5A–5C). Additionally, three more candidate genes selected from the enriched GO term of GO:0030833 — HNF1, CDC42 and alpha-actinin-4 — were also significantly increased (Supplementary Figure 5D–5F). Further, western blot showed that the protein levels of VDBP, VDR and GSN were significantly increased in MI-3d and MI-7d, consistent with the proteomic data (Supplementary Figure 5G). GAPDH was chosen as a housekeeping gene for both qRT-PCR and western blot.

**Figure 3:**
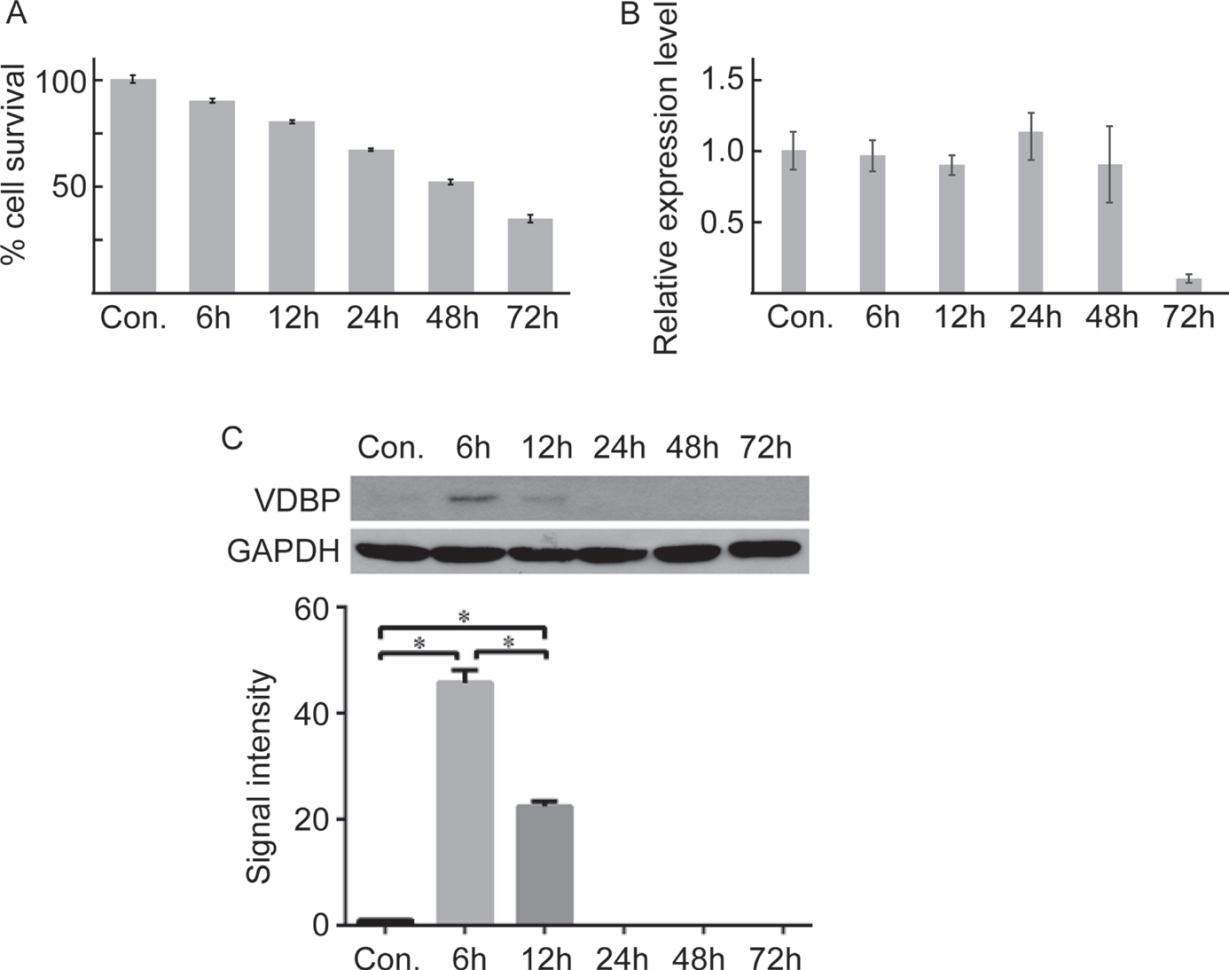
Quantitative PCR and western blot validation of VDBP in OGD/R cultured cells (each group, *n* = 5) (**A**) The cell survival rate at different times in OGD/R-cultured cells determined by MTS. (**B**) Real-time quantitative PCR of VDBP gene at different times after OGD/R culture. (**C**) Western blot (upper) and the gray signal intensities (lower) of VDBP for OGD/R-cultured cells at different times.

The oxygen and glucose deprivation/reperfusion (OGD/R) cell model was constructed using the H9C2 cell line to further validate the expression pattern of VDBP during acute myocardial infarction. The percentages of cell survival at 6 h, 12 h, 24 h, 48 h and 72 h after OGD/R treatment were decreased (Figure 3A). Cell apoptosis were determined by TUNEL and flow cytometry assays, showing similar results (Supplementary Figure 6). Interestingly, although the mRNA levels were not significantly changed, the protein levels of VDBP were significantly higher at 6 h and 12 h after OGD/R treatment (Figure 3B, 3C). The post-transcriptional regulation mechanism was not clear; however, the expression pattern of VDBP in both AMI model mice and the OGD/R-treated cell lined showed great potential for this protein to serve as an early diagnosis biomarker for AMI.

### Overexpression of VDBP promoted the cardiomyocyte apoptosis after hypoxia

**Figure 4:**
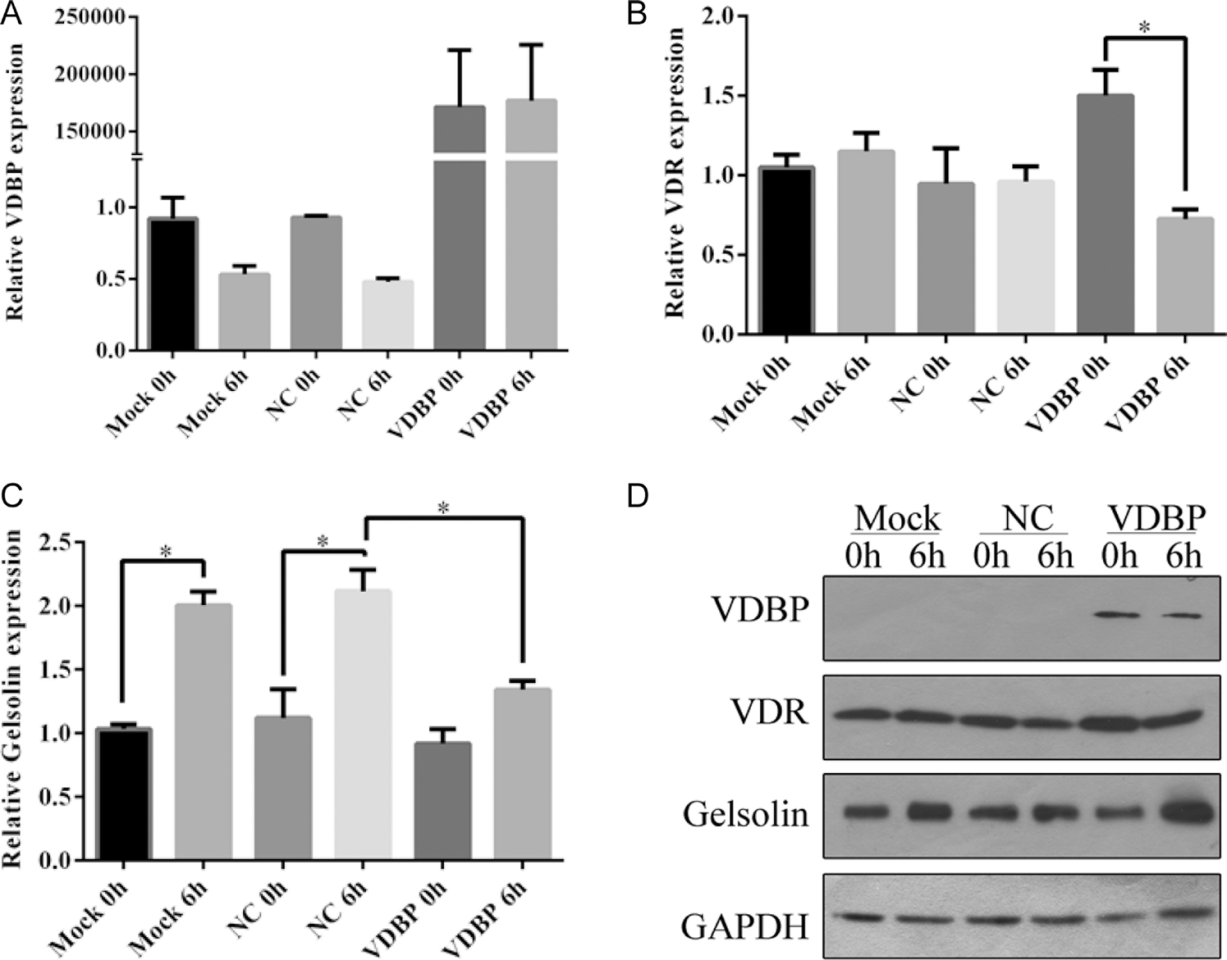
Overexpression of VDBP in H9C2 cells reducing the expression level of vitamin D receptor after OGD/R culture (each group, *n* = 5) Relative expression levels of VDBP (**A**), VDR (**B**) and GSN (**C**) were determined by quantitative PCR in H9C2 cells at 0 h and 6 h of OGD/R culture. Mock, standard H9C2 cells; NC, cells transfected with blank vectors as negative control; VDBP, cells transfected with VDBP overexpression vectors. The expression level of each gene in mock 0 h was set at 1. (**D**) Protein levels of VDBP, VDR and GSN were determined by western blot. GAPDH was used as a control.

Transient overexpression of VDBP in H9C2 cells resulted in 150,000-fold increased mRNA levels and significantly increased protein levels (Supplementary Figure 7). The mRNA expression levels of VDBP showed a slightly decreased pattern after 6 h of OGD/R in both mock negative controls (transfected with blank vector) and over-expression cell lines. However, mRNA levels of the VDR gene had no significant changes in mock and negative controls while being surprisingly decreased in VDBP overexpressed cell lines. Moreover, mRNA levels of GSN were significantly increased after OGD/R, while much lower expression was found in VDBP overexpressed cells (Figure 4A–4C). The protein levels showed similar changes in corresponding mRNAs, except for the GSN protein level at 6 h after OGD/R in VDBP overexpression cells (Figure 4D). GAPDH was used as a control for both qRT-PCR and western blot. Each assay was performed with three independent biological duplicates.

**Figure 5:**
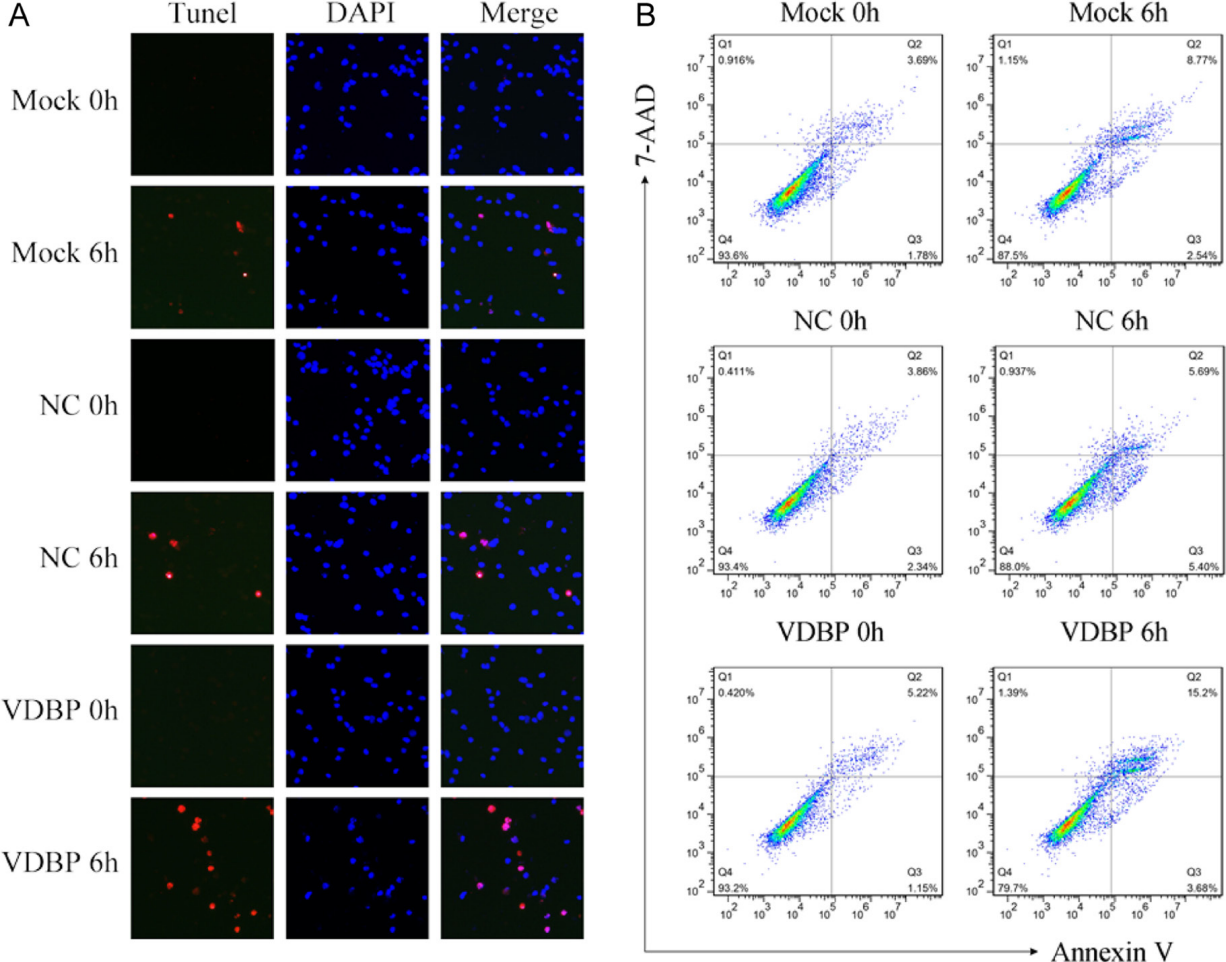
Overexpression of VDBP increased the OGD/R-induced cell apoptosis in H9C2 cells (each group, *n* = 5) Apoptosis was measured using TUNEL and flow cytometry assays in VDBP-overexpressed H9C2 cells. (**A**) TUNEL (red channel) assay showed the apoptosis of H9C2 cells, and DAPI (blue channel) was used to locate the nuclei of the cells. Merged images indicate cells only stained by DAPI. (**B**) Annexin V/7-AAD double-staining assay was used to quantify apoptosis rates in H9C2 cells by flow cytometry at 0 h and 6 h after OGD/R. Mock, standard H9C2 cells; NC, cells transfected with blank vectors as negative control; VDBP, cells transfected with VDBP overexpression vectors.

Further, cell apoptosis was examined by TUNEL and flow cytometry assays to assess the effects of overexpressing of VDBP. Cell lines overexpressing VDBP showed significantly increased cell apoptosis in TUNEL assay (red channel, Figure 5A). The annexin V/7-AAD double-staining flow cytometry assay showed that, compared to mock (8.77%) and negative controls (5.69%), significantly increased late apoptosis rate could be detected in VDBP overexpressed cells (15.2%, *p* < 0.001). Representative images are shown (Figure 5B).

## DISCUSSION

### iTRAQ data provided new candidate marker proteins for AMI

Proteomic investigations of AMI have mainly focused on new early potential biomarkers and mechanisms of remodeling after ischemia to seek more efficient diagnostic or treatment approaches [17, 24, 25]. Here, we identified 776 differentially abundant proteins after AMI, of which 128 were MI-3d special and 456 were MI-7d special. A total of 416 proteins were identified as AMI preferably accumulated proteins, of which 76 were MI-3d special, 210 were MI-7d special, and 120 were increased at both 3 and 7 days after AMI (Figure 2, Supplementary Tables 3–6). Several proteins in our list of DAPs have been reported to be crucial for AMI or left ventricular remodeling, including serum amyloid A protein (SAA) [26], S100 calcium binding protein A (S100A) [27], galectin [28], alpha-2-macroglobulin (A2M) [29], and cathepsin S [30]. Nevertheless, new candidates involved in the molecular changes after AMI might be identified by further examination of these data.

Gene ontology term enrichment analysis of the 120 proteins increased in both MI-3d and MI-7d showed that the top three significantly enriched branch-end GO terms were: blood coagulation (GO:0007596); complement activation (GO:0006956); and humoral immune response mediated by circulating immunoglobulin (GO:0002455) (Supplementary Table 7, Supplementary Figure 2). Not much attention has been paid to reduced proteins after AMI. However, our data provided GO term enrichment analysis for the 72 proteins reduced in both MI-3d and MI-7d (Supplementary Table 8, Supplementary Figures 3, 4). These pathways might be the majority of biological processes influenced by heart dysfunction after AMI. Further research work should be performed to investigate the mechanisms underlying how about these reduced proteins were involved.

### Vitamin D binding protein might be important during heart failure after AMI

The role of vitamin D in cardiovascular disease has not been clearly illustrated. The Atherosclerosis Risk in Communities Study (ARIC) showed that low serum 25(OH)D was independently associated with incident heart failure, and low 25(OH)D was associated with HF risk predisposal with high VDBP [31]. However, in a meta-analysis, the effect of vitamin D co-administered with other interventions did not provide effective evidence of vascular protection [32]. Another study reported that VDR might not be detectable in normal cardiac muscles [33]. In contrast, there was some strong evidence to show that the expression of VDR was increased in the hypertrophic heart [34–36].

The GO term enrichment results of molecular function categories showed that binding was the most significantly enriched term, including calcium ion binding, GTP binding, actin binding, lipid binding and cytoskeletal protein binding (Supplementary Figure 2). Thus, vitamin D binding protein, which carries vitamin D sterols and prevents polymerization of actin by binding to its monomers, was selected to perform further validation [37]. The expression levels of VDBP, as well as of VDR and GSN, were increased in AMI mouse tissue (Supplementary Figure 5). Moreover, VDBP was significantly increased shortly after AMI or OGD/R and then decreased to normal expression levels in mouse tissue or cell culture, consist with the iTRAQ results (Figure 3C). However, the mRNA levels of VDBP were not significantly changed after AMI, indicating that an un-clear post-transcriptional mechanism might be involved (Figure 3). Similarly, inconsistent levels of mRNA and proteins were found for GSN in VDBP overexpression cells (Figure 4C, 4D).

Overexpression of VDBP in H9C2 cells led to a significantly increased cell apoptosis rate, indicating that the accumulation of VDBP proteins in LV tissue might be important for dysfunction after AMI (Figures 3—5). Another interesting result was that both the mRNA and protein levels of VDR were decreased in VDBP overexpression cells after OGD/R treatment, indicating that there might be a negative feedback influence of VDBP on VDR (Figure 4B, 4D). Studies showed that VDBP was the main blood carrier of vitamin D, but active vitamin D worked only when it was unbound from protein carriers, which exert most of the biological functions via binding of vitamin D and VDR [38, 39]. Therefore, a high level of VDBP could decrease the activity of vitamin D and the level of VDR.

In summary, we investigated the proteome changes after AMI in a mouse model. Of the 2560 proteins identified, 776 were significantly differentially accumulated in either MI-3 or MI-7. The molecular function categorization of GO term enrichment showed that the molecular function of binding was the most significant. Quantitative PCR and western blot assays showed that vitamin D binding protein was increased in both AMI tissues and OGD/R H9C2 cells. Overexpression of VDBP in H9C2 cells significantly promoted cardiomyocyte apoptosis after hypoxia.

### Clinical perspectives

Our work investigated the proteome changes after AMI, providing a list of differentially abundant proteins (DAPs) that could potentially be considered novel biomarker candidates. Some of the DAPs were reported to be involved in AMI for the first time. Understanding the biological functions of these DAPs could help doctors to make more efficient diagnoses or to devise better treatment approaches for AMI.

### Translational outlook

Our data provided evidence that vitamin D binding protein might be involved in left ventricular remodeling. Overexpression of VDBP in H9C2 cells resulted in higher cell apoptosis after hypoxia. This finding indicated the possibility of VDBP serving as a medical target in AMI treatment. Scientists could artificially active or inhibit the function of VDBP to achieve better prognoses in AMI treatment.

## MATERIALS AND METHODS

All of the experiments were performed with the approval of the animal ethics committee of the First Affiliated Hospital of Xinjiang Medical University in accordance with the Care and Use of Laboratory Animals guidelines of the National Institutes of Health (permit number: IACUC-20141217012). Details of the methods are provided in the Online Supplementary Material.

### Mouse material and protein extraction

Male C57B/6J mice were randomly divided into 4 groups and housed in a facility with a 12–12-h dark-light cycle and free access to standard mouse chow and water. The mice were anesthetized by intraperitoneally administering a mixture of ketamine, xylazine, and atropine (100, 20, and 1.2 mg/kg ip, respectively) and were ventilated with a rodent ventilator (model 683; Harvard Apparatus). The left coronary arteries were ligated with a 7–0 silk suture at a level approximately 1 mm below the edge of the left auricle to construct an AMI model. Sham surgery was also performed without ligating the left coronary artery. The animals were inspected at least four times daily until death or sacrifice at day 3 (MI-3d, Sham-3d) and day 7 (MI-7d, Sham-7d). The infarcted area could be determined by its pale color, intramyocardial hemorrhages, and reduction in the wall thickness of the non-infarcted myocardium. Total protein was extracted from tissues as previously described [19]. At the end of the experimental protocol, the mice were sacrificed by intraperitoneally administering 1% pentobarbital (100 mg/kg), followed by cervical dislocation.

### iTRAQ and data processing

A Triple TOF 5600+ system (AB Sciex) was used to perform iTRAQ analysis, coupled with a Nanoflex microchip system (Eksigent, Dublin, CA, USA). Briefly, trypsin-digested protein samples from the Sham-3d (isobaric tags 113, 114), Sham-7d (isobaric tags 115, 116), MI-3d (isobaric tags 117, 118) and MI-7d (isobaric tags 119, 121) groups were labeled using iTRAQ Multiple Plex reagents (AB Sciex, No. 4352160). After labeling, the peptides were divided into 15 fractions by 2D LC MS/MS analysis as previously described [20].

Protein identification and relative iTRAQ quantification were performed using Protein Pilot software, version 5.0 (AB Sciex). In this study, a protein was considered identified only when the “unused” confidence threshold (Prot. Score of Protein Pilot software) > 1.3, and the corresponding FDR was less than 5%. The relative abundance of proteins was calculated based on individual peptide ratios, and the thresholds for differential abundant proteins were fold changes > 2 and *P* values < 0.05.

### Bioinformatic analysis

Gene ontology (GO) annotations of all of the identified proteins were determined by Blast2GO [21], using the Uniprot Accs. GO term enrichment analysis was performed using online tool agriGO [22].

### Cell culture and oxygen and glucose deprivation/reperfusion (OGD/R) model construction and measurement of cell apoptosis

An oxygen and glucose deprivation/reperfusion (OGD/R) model was constructed by replacing the culture medium with serum-free DMEM and inflating it 5% carbon dioxide and 95% nitrogen for cultured H9C2 cells. Cells were harvested at 6 h, 12 h, 24 h, 48 h and 72 h after OGD/R. Cell apoptosis was determined by TUNEL and flow cytometry assays as previously described [23]. Five independent experiments were performed.

### Statistical analysis

All of the data were analyzed using SPSS software, version 19 (SPSS Inc., Chicago, IL, USA) using one-way ANOVA or Student’s *t*-test. Statistical significance was set at *p* < 0.05.

Yun Wu, Fen Liu and Yi-tong Ma had full access to all of the data in the study and accept responsibility for the integrity of the data and the accuracy of the data analysis.

## SUPPLEMENTARY MATERIALS FIGURES AND TABLES




