# Supplementary material for: iTRAQ analysis of a mouse acute myocardial infarction model reveals that vitamin D binding protein promotes cardiomyocyte apoptosis after hypoxia
Source: Oncotarget. 2017 Dec 6;9(2):1969–79. doi: 10.18632/oncotarget.23025 (PMC5788613; doi:10.18632/oncotarget.23025)
Supplement: Supplementary file 1 [file oncotarget-09-1969-s001.pdf]

iTRAQ analysis of a mouse acute myocardial infarction model reveals that vitamin D binding protein promotes cardiomyocyte apoptosis after hypoxia

SUPPLEMENTARY MATERIALS

Sample size calculation

The sample size was based on a completely randomized multi-sample mean comparison calculation formula and the mortality of mice myocardial infarction model in previous experiment.

Echocardiographic and hemodynamic assessment

Transthoracic echocardiography was performed at day 3 and day 7, respectively, using a Hewlett-Packard Sonos 5500 ultrasound machine with a 15-MHz linear-array transducer, as described previously. The following parameters were measured and calculated: LV internal end-systolic and end-diastolic diameters (LVESd and LVEDd, respectively), external LV diastolic diameter (ExLVDd), anterior wall thickness at systole and diastole (Awsth and Awdth, respectively), and fractional shortening (FS % = [LVEDd- LVESd/ LVESd ×100%]).

Hemodynamics parameters were measured in sham-operated and infarcted mice. After anesthesia (as used for echocardiography), a 1.4-Fr Millar catheter was placed in the LV through the right carotid artery. Heart rate was obtained by pulse signals. The following parameters were measured with PowerLab Chart 4.1.2 software (AD instruments): systolic blood pressure (SBP), diastolic blood pressure (DBP), LV systolic pressure (LVSP), and the maximal rates of rise and fall in LV pressure (dP/dtmax and dP/dtmin, respectively).

During echocardiographic and hemodynamic assessment, Animals were lightly anesthetized with a half dose of the anesthetic mixture for surgery.

Quantitative real-time PCR analysis

Total RNA was extracted using Trizol reagent according to the product protocol (Invitrogen, Cat# CW0581). For reverse transcription, 1 µg of total RNA was converted to first-strand cDNA in a 14.5-µl reaction mixture using the TIANScript RT reagent Kit (TIANGEN, Beijing, China, Cat# KR104-02). Quantitative real-time PCR analysis was performed using an ABI 7500 fluorescence quantitative PCR instrument (Applied Biosystems) using the $2^{-\Delta\Delta CT}$ method. The thermal cycling program began with 10min at 95 °C for enzyme activation and then included 40 cycles of denaturation for 10 s at 95°C, 30-s annealing at 58 °C, and 30 s extension at 72°C. Primer sequences are listed in Supplementary Table 9.

Western blot

To assess protein levels, total protein was extracted from stored infarcted area of left ventricular tissues. Next, protein extracts were separated by 10% SDS-PAGE and transferred to polyvinylidene difluoride (PVDF) membranes (pore diameter 0.22 µm). The membranes were blocked at room temperature for 1 h with a blocking solution (3% BSA) in TBST buffer (10 mM Tris, 100 mM NaCl, 0.1% Tween 20, pH 7.4). The blots were incubated overnight at 4°C with primary antibodies, including anti-VDBP (Abcam, Product code ab153922), anti-VDR (Abcam, Product code ab8756), anti-GSN (Abcam, Product code ab74420). The membranes were then washed in TBST buffer 3 times for 10 minutes each. Bound primary antibodies were incubated with horseradish peroxidase-labeled secondary antibodies for 1 h at room temperature, washed, and detected using ECL. Densitometry was conducted with a UVP gel imaging system (USA). Blots were stripped and re-probed with a GAPDH antibody (Abcam, Product code ab8245) to confirm equal loading.

Transientoverexpression of VDBP

The full length 1,431 bp ORF of VDBP was synthesized (Generay, Shanghai, China)with*Xba*I/*Not*I restrict sites and cloned into overexpression vector LV003.Cells were incubated for 3 hours with vectors overexpressing VDBP (blank vector was used in mock experiment). Transienttransfection was performed usingLipofectamine® 3000 (Thermo Fisher Scientific, USA) following the manufacture’s construction. Cells were then washed and centrifuged to obtain pellets after transfection. Overexpression of VDBP was verified at gene (qPCR) and protein (western blot) level, respectively.

Supplementary Table 1: Body weight and echocardiographic measurements before surgery

Parameters*	Sham-3d (*n* = 10)	MI-3d (*n* =10)	Sham-7d (*n* =10)	MI-7d (*n* =10)	*P*#
BW(g)	26.46 ± 2.27	27.31 ± 1.44	27.49 ± 1.97	27.08 ± 2.61	0.712
LVEDd (mm)	3.31 ± 0.12	3.28 ± 0.26	3.40 ± 0.13	3.24 ± 0.42	0.733
LVESd (mm)	1.66 ± 0.28	1.61 ± 0.31	1.78 ± 0.27	1.52 ± 0.30	0.22
ExLVDd (mm)	4.81 ± 0.38	4.67 ± 0.39	4.96 ± 0.47	4.71 ± 0.50	0.443
LVAWs (mm)	1.43 ± 0.12	1.41 ± 0.11	1.43 ± 0.14	1.34 ± 0.15	0.283
LVAWd (mm)	0.85 ± 0.13	0.83 ± 0.16	0.86 ± 0.17	0.79 ± 0.12	0.782
FS (%)	49.80 ± 6.26	50.94 ± 8.22	47.48 ± 7.91	53.18 ± 6.70	0.325

*BW, body weight; LVEDd, left ventricular internal end-diastolic diameters; LVESd, left ventricular internal end-systolic diameter; ExLVDd, external left ventricular diastolic diameter; LVAWs, left ventricular systolic anterior wall thickness; LVAWd, left ventricular diastolic anterior wall thickness; FS, fractional shortening.

#One-way ANOVA *p* value.

Supplementary Table 2: Statistics of mass spectra data*

Cutoff P value	Total spectra	Identified spectra	Distinct spectra	Proteins before grouping	Detected proteins
0.05	545987	269526	61818	12451	2540
0.01	545987	265731	59862	10878	1925

Supplementary Table 3: Identification and quantitative information of 196 proteins that significantly accumulated at MI 3d compared to Sham 3d. See Supplementary_Table_3

Supplementary Table 4: Identification and quantitative information of 124 proteins that significantly reduced at MI 3d compared to Sham 3d. See Supplementary_Table_4

Supplementary Table 5: Identification and quantitative information of 330 proteins that significantly accumulated at MI 7d compared to Sham 7d. See Supplementary_Table_5

Supplementary Table 6: Identification and quantitative information of 328 proteins that significantly reduced at MI 7d compared to Sham 7d. See Supplementary_Table_6

Supplementary Table 7: GO term enrichment analysis of proteins that are preferably accumulated in both MI-3d and MI-7d (FDR < 0.05). See Supplementary_Table_7

Supplementary Table 8: GO term enrichment analysis of proteins that are preferably reduced in both MI-3d and MI-7d (FDR < 0.05). See Supplementary_Table_8

Supplementary Table 9: Primers used in this work

Gene name	Forward primers (5'-3')	Reverse primers (5'-3')	Product size (bp)
Mus musculus			
GAPDH	AGAAACCTGCCAAGTATGATGAC	GAGGCCATGTAGGCCATGAG	250
VDBP	GACACCGTGGAGGTGCTAAT	TACTTCTCATCCTTCTGTTCCTCAG	185
VDR	GCTCATGGCCATCTGCATTG	CCTCTAGCACAAGGGGTGTG	266
GSN	TGTGCAGCCTGTAAGCCCAG	CCGGCCGTTCAGGTAGTCAT	297
HNF1 homeobox A	AAGAGGTAATCCGGCAGGG	CTCTTTGCTCAGGCCAGACT	292
HNF1 homeobox B	AGGAGAGGAGCCCAGACTCTA	CTGTCGGAGGATCTCCCGTT	229
CDC42	CCAGAGACTGCTGAAAAGCTG	AGATGCGTTCATAGCAGCAC	170
α-actinin-4	GGCCCTCATCTTCGACAACA	CCGGTCATTCTCCACGTCAT	268
β-actin	AGTGTGACGTTGACATCCGT	AGCTCAGTAACAGTCCGCCTA	296
Rattus norvegicus			
GAPDH	TATGACTCTACCCACGGCAAG	ATACTCAGCACCAGCATCACC	138
VDBP-qPCR	GGGAAACAGCAAGCCCAAAG	GTAGCGTGAAAGCAGGGACC	166
VDR	CCATTCAGGACCGCCTATCC	AGGAGAGGGAGCGGTATTGT	156
GSN	CCCCAAAGTCGGGTGTCTGA	CTCGTTGCCCAGCCAATAGT	240

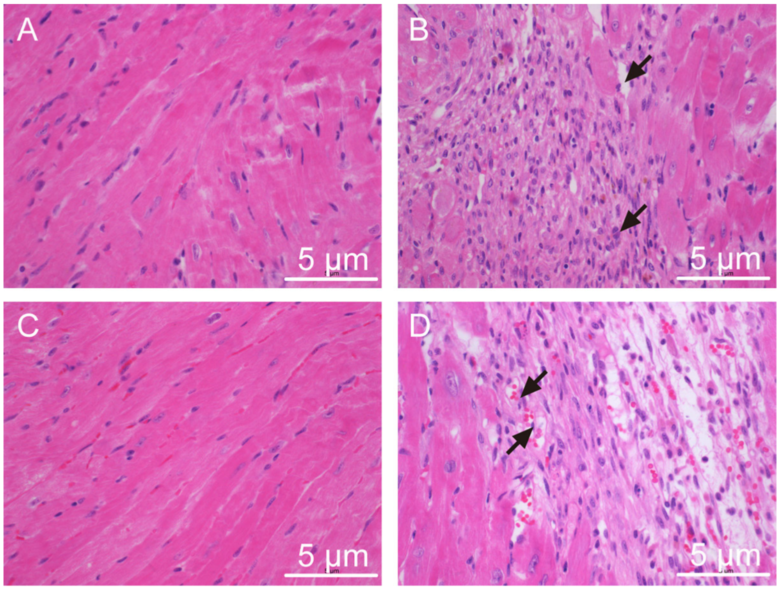

Supplementary Figure 1: Hematoxylin and eosin (H&E) staining images of heart tissue samples from 4 groups. Myocardial histology examples from the sham-3d (**A**), MI-3d (**B**), sham-7d (**C**), MI-7d (**D**) are shown. In H&E staining images, the cytoplasm is red and the nucleus is blue. In sham-3d and sham-7d groups (A and C), myocardial fibers were cylindrical in an orderly arrangement. In MI-3d group (B) compared with sham-3d group (A), coagulative necrosis of myocardial fibers, myocardial interstitial edema, and lots of inflammatory cells infiltration were shown in MI area. In MI-7d group (D), new capillaries granulation tissue proliferation was observed in MI area. Scale bars = 5 μm.

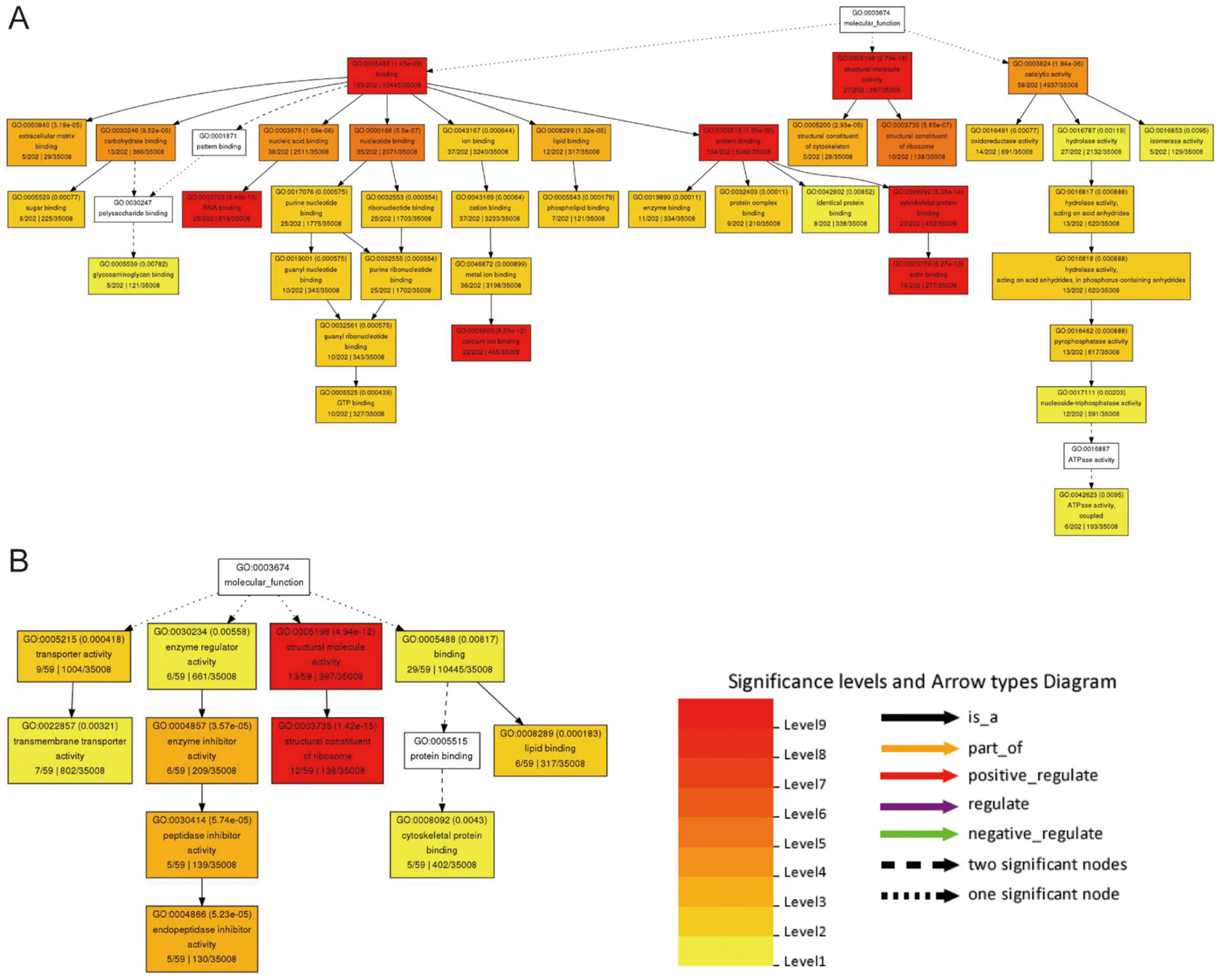

Supplementary Figure 2: GO term enrichment analysis of MI-7d specially accumulated proteins (**A**) and MI-3d specially accumulated proteins (**B**) by agriGO. GO terms which were significantly enriched (FDR *p* value < 0.01) were shown in colored boxes. The significance levels and meaning of arrow types were indicated.

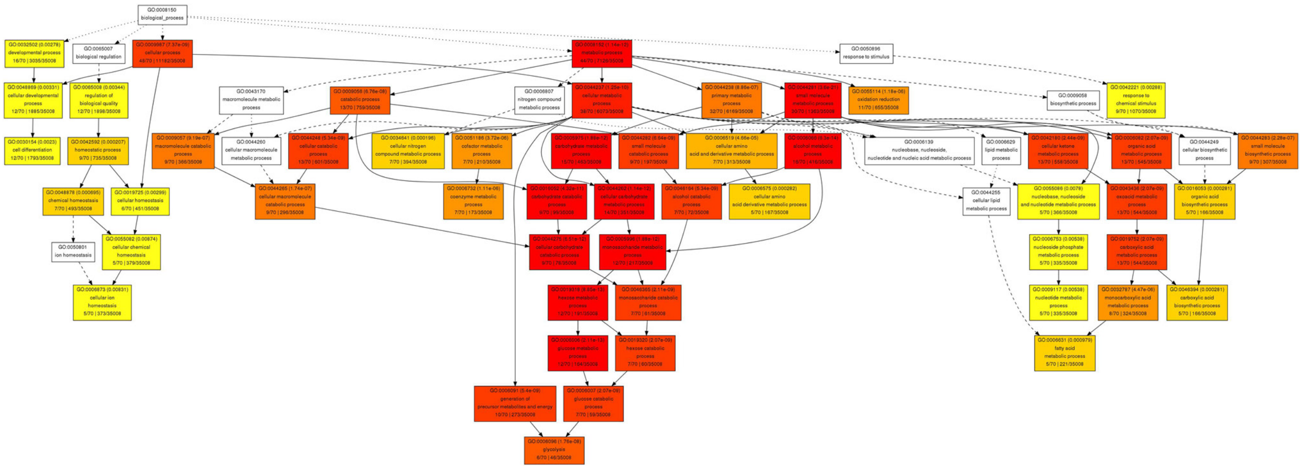

Supplementary Figure 3: GO Term of biological process enrichment analysis of protein reduced in MI-3d. The arrows and the color saturation of the boxes represented the same meaning as in Supplementary Figure 2.

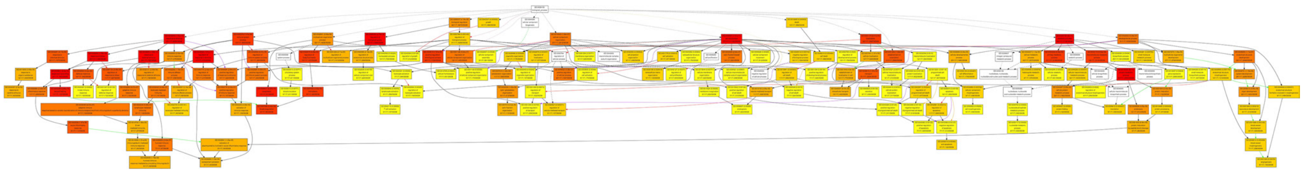

Supplementary Figure 4: GO Term of biological process enrichment analysis of protein reduced in MI-7d. The arrows and the color saturation of the boxes represented the same meaning as in Supplementary Figure 2.

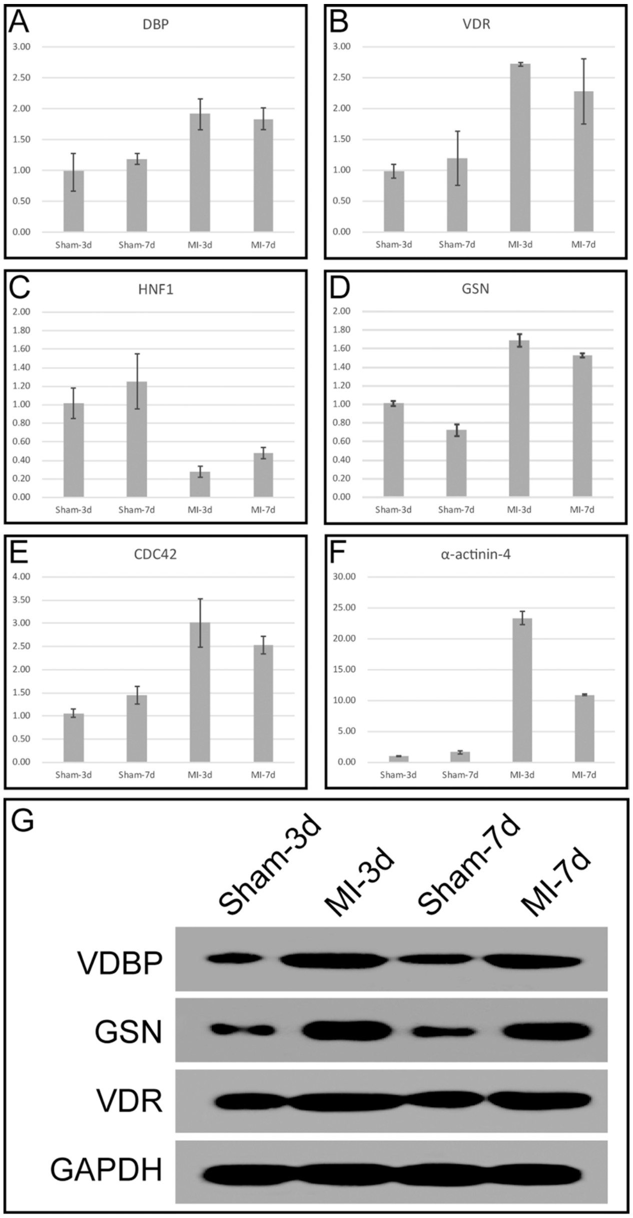

Supplementary Figure 5: Quantitative PCR and Western blot validation of representative differentially accumulated proteins. Real-time quantitative PCR of vitamin D related genes were shown in (**A–D**). DBP, vitamin D binding protein; VDR, vitamin D receptor; HNF1, hepatic nuclear factor 1; GSN, gelsolin. And two representative genes selected from enriched GO terms GO:0030833 were shown in (**E** and **F**). CDC42, cell division cycle 42; a-actin-4, actinin alpha 4. The relative expression level of each gene in Sham-3d was set to 1 (n = 5). (**G**) Western blot of three vitamin D related proteins in Sham-3d, Sham-7d, MI-3d and MI-7d.

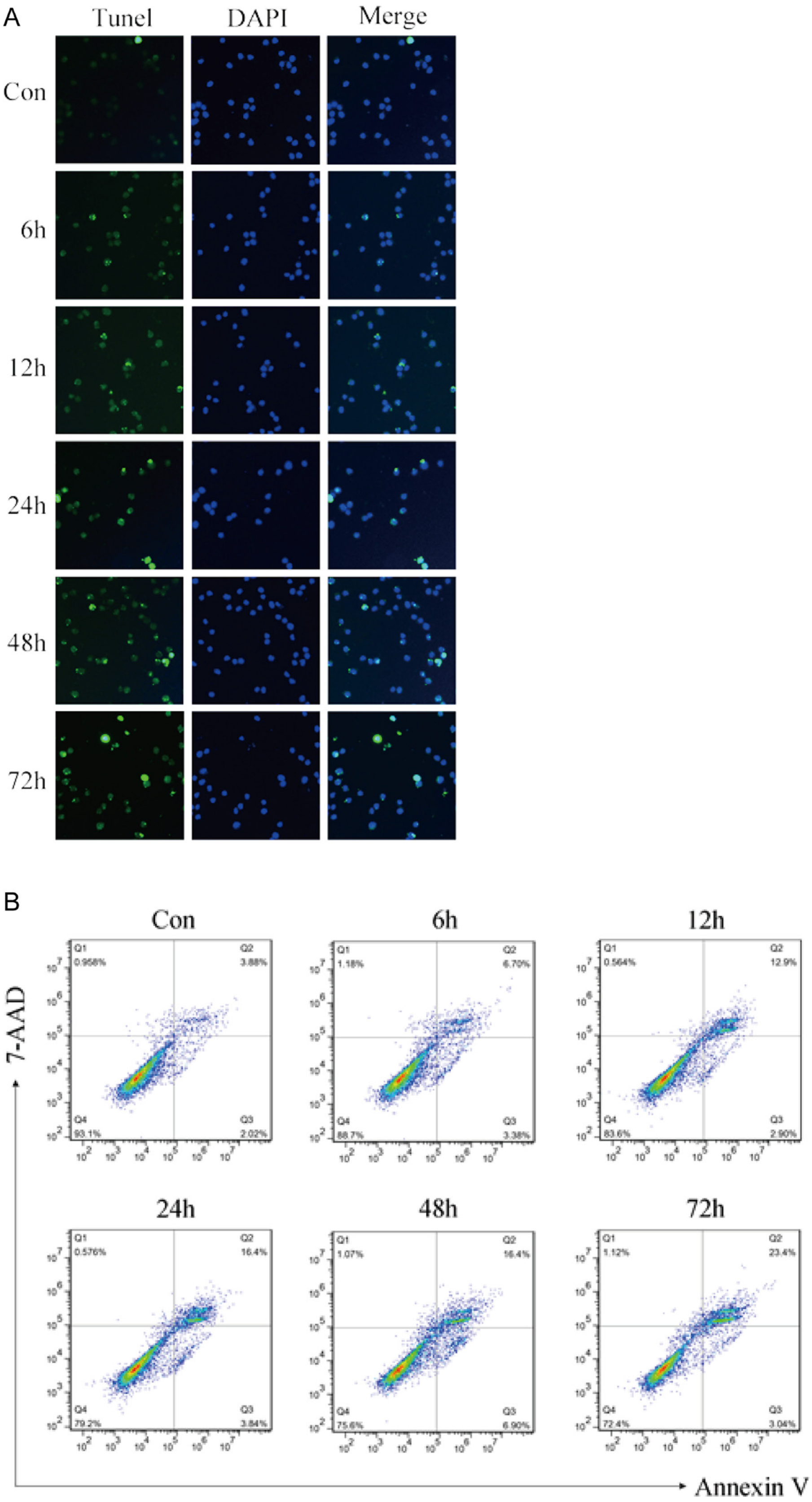

Supplementary Figure 6: TUNEL and flow cytometry assays of OGD/R cultured H9C2 cells at different time.Apoptosis was measured using TUNEL and flow cytometry assays in H9C2 cells after OGD/R. (**A**) TUNEL (green channel) assay showed the apoptosis of H9C2 cells and DAPI (blue channel) was used to locate the nuclei of the cells. Merged images indicated cells only stained DAPI. (**B**) Annexin V/7-AAD double-staining assay was used to quantify apoptosis rates in H9C2 cells by flow cytometry at 0 h, 6 h, 12 h, 24 h, 48 h and 72 h after OGD/R.

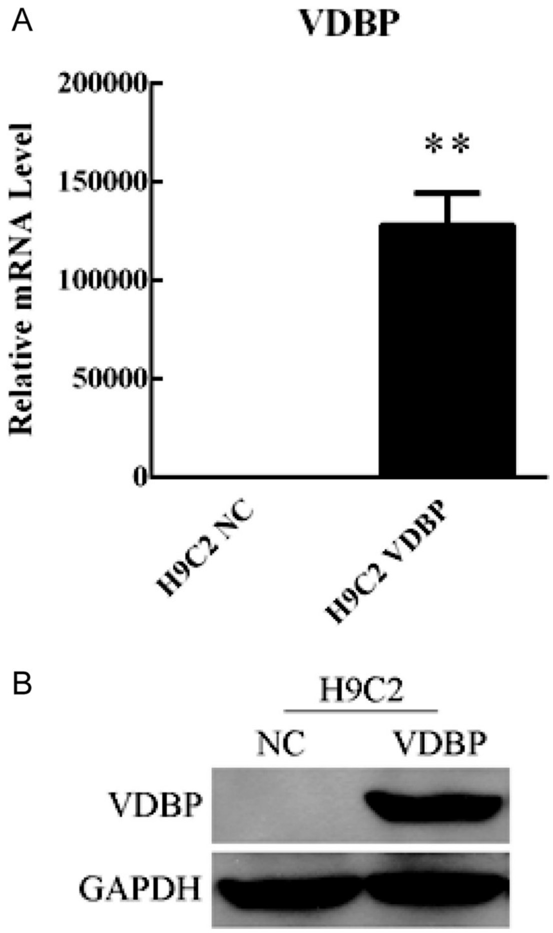

Supplementary Figure 7: Quantitative PCR (**A**) and Western blot (**B**) Validation of VDBP overexpression intransient transfection H9C2 cells.
